# Epithelioid Hemangioma of the Popliteal Artery: A Case Report

**DOI:** 10.7759/cureus.56011

**Published:** 2024-03-12

**Authors:** Amaury Alexander Martinez, Gagan Sathya Prakash, Mansi Sanghvi, Jigyasha Pradhan, Hanasoge Girishkumar

**Affiliations:** 1 Surgery, BronxCare Health System, New York, USA; 2 Vascular Surgery, BronxCare Health System, New York, USA

**Keywords:** great saphenous vein grafting, endothelioid hemangioma, popliteal artery aneurysm, vascular bypass, epitheliod hemangioma

## Abstract

An epithelioid hemangioma (EH) is a rare benign vascular lesion that is usually seen in superficial small vessels within the dermis and subcutaneous tissue. Intravascular epithelioid hemangiomas of large and medium-sized vessels are rare, and only a handful of cases have been reported in the literature. Intravascular epithelioid hemangiomas are biologically benign and best treated by complete surgical excision. On occasion, lesions have been associated with aneurysmal changes in the affected vessel. Local recurrence may occur, and close clinical follow-up is advised. Herein, we report the second case in the literature of an EH originating from the popliteal artery. A 57-year-old male patient presented with a one-month history of knee pain without claudication. Imaging highlighted a right popliteal aneurysm, 5x5 cm, with partial distal thrombosis and inadequate outflow. The patient subsequently underwent popliteal artery ligation above and below the aneurysm, reconstructed with a superficial femoral artery (SFA) to distal anterior tibial artery (ATA) reverse saphenous vein bypass graft. Patient recovery was complicated by the development of a 5x5 cm right-sided mid-thigh hematoma, requiring evacuation under anesthesia. A post-one-year arterial duplex of the affected limb demonstrated a recurrent enlarging popliteal aneurysm measuring 5.7x4.8x9.1 cm. The aneurysm was reported to be mostly thrombosed with noted vascularity, but patency of the original bypass was noted. The patient underwent excision of the recurrent aneurysm with subsequent ligation of the feeding arteries. Pathology and histology confirmed the final diagnosis of EH of the popliteal artery. An 18-month follow-up after the excision procedure demonstrated no recurrence of vascular lesion and patency of the original bypass graft.

## Introduction

An epithelioid hemangioma (EH), also known as angiolymphoid hyperplasia with eosinophilia (ALHE), is a rare benign vascular lesion of uncertain pathogenesis most commonly affecting superficial small vessels within the dermis and subcutaneous tissue. EH typically presents as a dermatologic small superficial vascular lesion of bright or dusky red papules or nodule [1-3]. EH incidence is greater in females than males and is more prevalent in the Asian female population during the third and fourth decades of life [1,4,5]. 

Although epithelioid hemangiomas are usually located in the distal extremities or within the head and neck region, rare cases of intravascular epithelioid hemangiomas of large and medium-sized vessels have been previously reported [3]. Deep epithelioid hemangiomas are atypical but are usually biologically benign and best treated by complete excision. Occasionally, lesions have been associated with aneurysmal changes in the affected vessel. Local recurrence may occur; therefore, intensive clinical follow-up is recommended. 

Here we report the second case in the literature of an EH in the popliteal artery, in which the patient presented with a right popliteal aneurysm with intra-mural thrombus, undergoing superficial femoral artery (SFA) to distal anterior tibial artery (ATA) reversed saphenous vein bypass with excision of the right popliteal artery aneurysm and ligation of feeding arteries.

## Case presentation

A 57-year-old Hispanic man presented to the clinic with persistent posterior right knee pain for a month. The pain was progressive. He denied arterial claudication, rest pain, tissue loss, or prior trauma. The patient was a nonsmoker, and his past medical history was significant for hypertension and prostate adenocarcinoma, for which he underwent a robotic prostatectomy.

**Figure 1:**
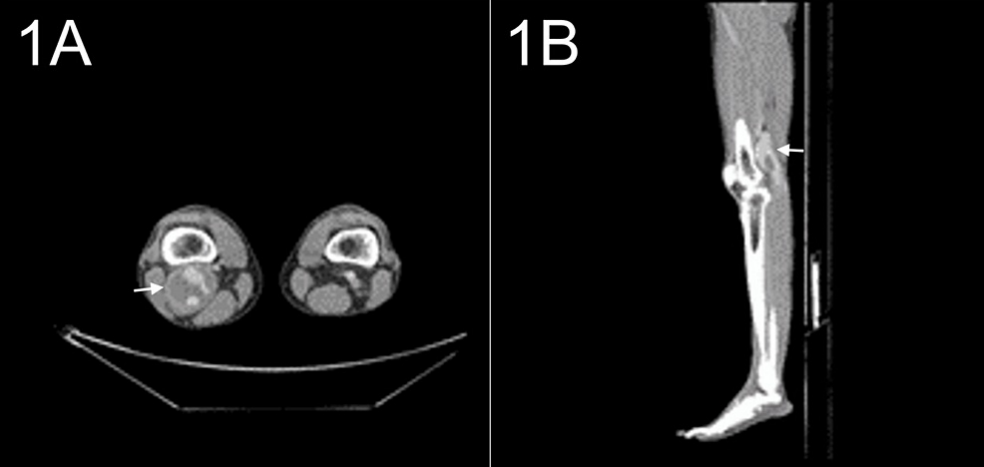
CT angiogram of lower extremities CT angiogram of the patient's lower extremities identified a 5x5 cm popliteal artery aneurysm (1A) and distal thrombosis, leaving inadequate outflow into the tibioperoneal trunk (1B).

On physical examination, the patient had palpable femoral pulse 2+, popliteal 2 +, and pedal pulses 1+. There were no associated masses, skin changes, or edema. Laboratory tests did not reveal peripheral eosinophilia, and IgE was not measured. Duplex ultrasound reported a right popliteal artery aneurysm with mural thrombus. A subsequent CT angiogram (CTA) reported a right popliteal aneurysm of 5x5 cm (Figure 1A) with partial distal thrombosis and inadequate outflow into the tibioperoneal trunk (Figure 1B). However, distal reconstitution was appreciated in the anterior tibial artery (ATA). CT angiogram of the abdomen and chest ruled out concurrent aneurysms and gross atherosclerotic changes. Cardiac valvular vegetation was also ruled out with an echocardiogram.

Following CTA, the patient was planned for an angiogram for further evaluation, and the results were compatible with the CTA findings. It was found that the ATA was the single vessel runoff to the foot and was reconstituted via collaterals.

As initial operative management, the patient underwent popliteal artery ligation above and below the aneurysm, and a reverse saphenous vein bypass graft was utilized to revascularize from the SFA to the distal ATA. The procedure was uneventful, but the postoperative period was complicated with the development of a hematoma, 5x5 cm, within the right mid-thigh on postoperative day 13, which required surgical intervention, and the patient was taken back to the operating room for evacuation under anesthesia, after which patient was discharged with scheduled follow up in the clinic.

**Figure 2:**
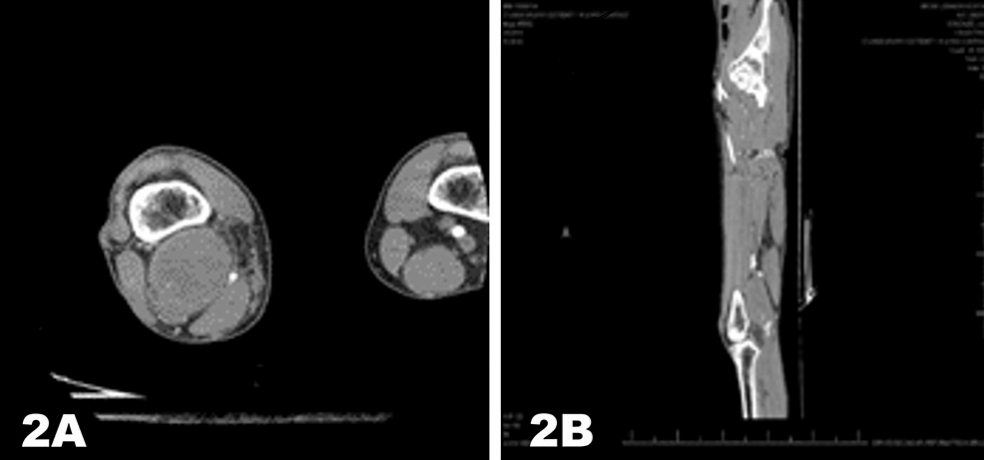
Repeat CT angiogram of lower extremities CT angiogram of the lower extremities demonstrating an enlarging thrombosed popliteal aneurysm in the left popliteal artery (2A) with patent flow through the reverse venous bypass graft (2B).

During the postoperative follow-up period, the right lower extremity arterial duplex, as part of surveillance after one year, revealed an enlarging popliteal aneurysm, now measuring 5.7x4.8x9.1 cm (Figure 2A). The aneurysm was reported as mostly thrombosed, but vascularity was noted in the thrombus with a patent bypass graft (Figure 2B); follow-up CT confirmed the findings. 

Based on the imaging findings, further surgical intervention was pursued, this time electing for excision of the right popliteal artery aneurysm, intraoperatively three feeding arteries were noted which were ligated. The aneurysm was found to be 6x8 cm.

**Figure 3:**
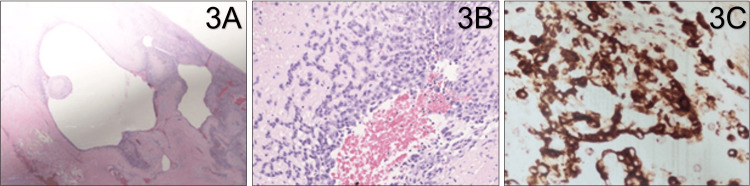
Epithelioid hemangioma Cross section of the aneurysm (H&E stain, 5x magnification) showing vascular spaces with myointimal hyperplasia, intraluminal thrombus material and granulation tissue (3A). Prominent epithelioid endothelial cell hyperplasia and focally invading the thrombus on H&E staining on 20x magnification (3B). Prominent epithelioid endothelial cells hyperplasia highlighted by immunostaining for CD31 (3C).

Postoperative pathology and microscopic analysis identified arterial wall focal granulation with hemosiderin pigment, myointimal hyperplasia, and prominent intraluminal thrombus material (Figure 3A). There was a proliferation of bland capillary-sized vessels (highlighted with a CD34 immuno-stain) that involved the periphery of the aneurysmal wall and focally penetrated its wall (Figure 3B). In many areas, the bland vessels were embedded in a dense inflammatory infiltrate that was rich in eosinophils and that transitioned to variably sized vessels with prominent large epithelioid endothelial cells that were also CD31, pankeratin, and CAM 5.2 positive (Figure 3C). Additional stains performed showed positivity for erythroblast transformation-specific (ETS)-related gene (ERG) in the lesioned cells and negativity for human herpesvirus 8 (HHV8) and NKX3.1. In addition, antigen Kiel 67​​​​​​​ (Ki-67) labeled many of the lesioned epithelioid cells.

The same large epithelioid cells proliferated and invaded the thrombus, focally reminiscent of a Masson's tumor or intravascular papillary endothelial hyperplasia. Additionally, the thrombus was lined with multiple cystically dilated luminal spaces. Histological analysis revealed atypical epithelioid cells with abundant amphophilic cytoplasm, large vesicular nuclei, and prominent nucleoli. However, the atypical cells were limited to the inner portion of the lesion without infiltrative growth, with limited mitotic activity. Overall, this constellation of histological findings was consistent with a final diagnosis of epithelioid hemangioma, with involvement of the popliteal artery and consequent secondary symptomatic-aneurysm development.

The patient was followed for 18 months post-excision. Multiple surveillance duplex ultrasounds during this period demonstrated patent bypass graft without signs of recurrence.

## Discussion

Epithelioid hemangiomas were first described in 1969 by Wells and Whimster [8,9]. An EH typically presents dermatologically with bright or dusky red papules or nodules, significant of small superficial vascular lesions. Histologically, EH is characterized as a lobular mass of small capillary-sized vessels surrounding a larger parent vessel, lined with histiocyte-like endothelial cells protruding into the vessel lumen surrounded by lymphoid germinal centers, lymphocytes, and eosinophils [4,9]. This unique histological appearance distinguishes the lesion from other hemangiomas during the differential diagnosis, especially when compared with Masson's hemangioma or Kimura disease. 

Masson's hemangioma is a benign vascular tumor that can also demonstrate papillary projections from the vascular wall on imaging due to the presence of exuberant intravascular epithelial hyperplasia. However, Masson's hemangioma commonly originates from a vein and is almost always associated with an organizing thrombosis. Conversely, Kimura disease is a chronic inflammatory condition with similar clinical and histopathological presentation to EH, differentiated by the absence of histiocytoid-epithelioid cells that are characteristic of EH [8].

The origins of EH are unclear, but have been hypothesized to be consequent to prior trauma, hyperestrogenemia, infectious etiologies, atopy, reactive hyperplasia, or benign neoplasms [10]. The lesion is considered a low-grade neoplasm due its minimal potential for progression [5,9].

Epidemiologically, EH is most commonly diagnosed in Asian females, 30 to 40 years of age. Anatomically, it appears in the dermis or subcutaneous tissue of the head and neck region. However, EH presentation has been reported in the medium and large vessels, bone, tongue, periosteum, and heart [8]. Patients with EH may be asymptomatic or may present with pruritus, pain, or spontaneous bleeding [10]. In 5-10% of patients, EH presents with regional lymphadenopathy or peripheral eosinophilia [5]. About half of the reported patients have multiple EH at once, generally within the same region. Recurrence is observed in one-third of the reported patients, though virtually no cases have given rise to metastasis [4].

In this case, the patient presented with an EH originating in the right popliteal artery. This is both rare in anatomical location and in size of the vessel involved. EH involving the coronary vessels, radial artery, brachial artery, popliteal artery, internal carotid artery, and occipital artery have been previously reported [4,6,7,9]. Abrahim et al. reported a case of EH originating from the internal carotid artery [3].

We conducted our own literature review of articles on PubMed that mention EH or ALHE of the popliteal artery. The year of publication was not limited as the intent was to view all previously existing cases. Only one previous case of histopathological proven epithelioid hemangioma of the popliteal artery has been reported by Ghotbi et al. [6], which described a 37-year-old patient with an epithelioid hemangioma arising from the third segment of the popliteal artery and the tibio-peroneal trunk. Herein, we report the second such case of EH in the popliteal artery. EH arising from other medium to large vessels, including internal carotid artery, carotid artery, radial artery, brachial artery, and occipital artery, have been described [3,4,7,9].

Various treatment methods for epithelioid hemangioma have been cited, including steroid injections, radiotherapy, cryotherapy, laser therapy, and excision. The variety of treatment options attempted reflects a knowledge gap in the pathogenesis of EH. Treatment failure, defined as the incomplete resolution of disease or recurrence after treatment, was lowest in cases treated with surgical excision (40.8%). Failure rates in other treatment modalities included pulsed dye laser (50%), carbon dioxide laser (54.6%), intermediate argon-laser therapy (66.7%), intralesional corticosteroids (79.1%), cryotherapy (80.5%), systemic corticosteroids (87.8%), and topical-only corticosteroids (98.2%) [10].

Surgical excision is considered the optimal treatment choice [8,10]. Intravascular presentation of EH is characterized by the formation of an arterial aneurysm, as seen in this case [6]. As such, it must be bypassed and/or removed when obstructing large vessels. However, surgical excision of EH involving medium or large vessels presents the risk of massive intraoperative bleeding. Therefore, proper preoperative angiography and embolization of feeding vessels can minimize intraoperative bleeding and confirm the existence of collateral blood supply to distal organs dependent on blood supply from vessels susceptible to transection during the excision [1]. Although EH is classified as a benign vascular tumor per the World Health Organization's (WHO) 2002 Classification [5], reported recurrence rates range between 33% and 40% [8,10]. This relatively high recurrence rate can be attributed to incomplete resection due to the difficulty of identifying the margins of a highly vascularized lesion [8,10]. Therefore, preoperative MRI with MR angiography should be utilized to assist in preoperative planning to facilitate complete surgical resection [8].

## Conclusions

Intravascular presentation of EH manifests as an arterial aneurysm, as seen in this case. Surgical excision is considered the optimal treatment choice for in these cases. Complete excision usually requires either bypass or removal of large vessel obstructions. Proper preoperative angiography and embolization of feeding vessels allow for the reduction in intraoperative bleeding and confirmation of existing collateral blood supply to the distal organs dependent on the pathological vessels requiring transection or excision.
